# Leflunomide in the Treatment of a Pseudotumoral Genital Herpes Simplex Virus Infection in an HIV Patient

**DOI:** 10.1155/2017/1589356

**Published:** 2017-03-08

**Authors:** Marie R. Roger, Gregory M. Anstead

**Affiliations:** ^1^IDT Clinic, 1905 Clint Moore Rd, Suite 305, Boca Raton, FL 33496, USA; ^2^Department of Medicine, Division of Infectious Diseases, South Texas Veterans Healthcare System, 7400 Merton Minter Blvd, San Antonio, TX 78229, USA; ^3^Department of Medicine, Division of Infectious Diseases, University of Texas Health Science Center at San Antonio, 7703 Floyd Curl Dr, San Antonio, TX 78229, USA

## Abstract

The patient is a 52-year-old African American man with a past medical history of HIV infection (on antiretroviral therapy, CD4 count 399 cells/*µ*L, and undetectable HIV viral load) and recurrent genital herpes. While on valacyclovir, the patient presented with four tumorous lesions on the perineum and scrotum. A biopsy specimen stained positively with HSV-1 and HSV-2 immunostains and displayed a lymphoplasmacytic infiltrate. The patient received foscarnet and imiquimod for two weeks with minimal improvement. Based on the previous activity of leflunomide, which has both antiviral and immunomodulatory properties, in cytomegalovirus and herpes simplex infections, leflunomide 20 mg orally twice daily was started. The patient received 23 days of foscarnet, 14 days of topical imiquimod, and 11 days of leflunomide with approximately 80% reduction in the size of the perineal lesion. After nine months on leflunomide there was complete regression of the large perineal lesion and only two small ulcerations remained on the scrotum. Pseudotumoral herpes lesions in HIV patients represent an immune reconstitution event and are poorly responsive to the usual anti-herpes agents. This report demonstrates the successful use of leflunomide in the treatment of an HIV patient with pseudotumoral herpes. Thalidomide has also been used with some success.

## 1. Introduction

Herpes simplex virus type 2 (HSV-2) is usually acquired by sexual contact and most commonly causes painful anogenital ulcerations. In patients with human immunodeficiency virus (HIV) infection, herpetic lesions can be persistent, multifocal, and more likely to recur [1]. Herpes simplex infections can also have atypical presentations in immunocompromised patients, including hypertrophic, verrucous, or pseudotumoral forms [1–6]. Herein, we present a case of an HIV-infected patient with recurrent mixed HSV-1/HSV-2 infection with pseudotumor formation that was poorly responsive to traditional antiherpes agents. The dermatologic and histopathologic features, as well as a significant response to treatment with leflunomide, an antiviral and immunomodulatory agent, are described.

## 2. Case Presentation

**Figure 1:**
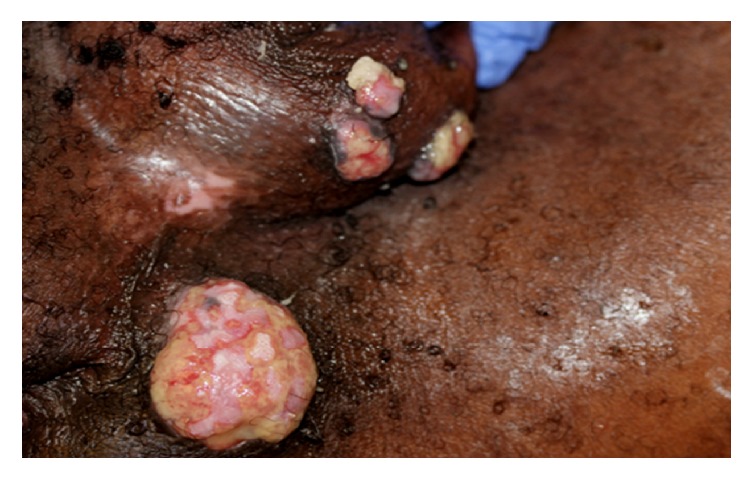
Appearance of tumorous lesions on the scrotum and perineum on admission.

The patient is a 52-year-old African American man with past medical history of HIV infection,* Mycobacterium avium* complex (MAC) infection, seizure disorder, and recurrent genital herpes. The patient was receiving antiretroviral therapy (ART) with emtricitabine/tenofovir and ritonavir-boosted darunavir, with a CD4 count of 399 cells/*μ*L (normal range 404–1612/*μ*L) (22%; normal range 33–58%) and an undetectable HIV viral load. He was admitted for intravenous foscarnet therapy of a recurrent genital herpes infection that was unresponsive to oral valacyclovir. His first episode of genital herpes had occurred three years prior to admission; he was treated at that time with valacyclovir (1000 mg twice daily for 14 days) with complete resolution. However, there was a recurrence about 18 months later for which he received oral valacyclovir (1000 mg twice daily for 14 days) with nearly complete resolution of the lesions. Acyclovir (400 mg po bid) was maintained for chronic suppressive therapy; however, three months later he had another recurrence. Intravenous cidofovir was administered for four weeks (three doses), but there was no significant improvement in the ulcerative lesions so he was switched to foscarnet. The patient completed a 21-day treatment with foscarnet along with topical imiquimod with partial response. He was placed back on valacyclovir (1000 mg orally twice daily) for ongoing treatment of herpes. After four months of valacyclovir treatment, he presented with four tumorous lesions in the perineal and scrotal areas. Physical exam was unremarkable except for three exophytic, nontender nodular lesions, about 1-2 cm in diameter, along the left side of the scrotum and a 5 cm in diameter mass in perineal region (Figure 1). Initial routine laboratory results (complete blood count and comprehensive metabolic profile) were within normal limits. He was started on intravenous foscarnet 40 mg/kg twice a day in addition to topical imiquimod. Due to concern for malignancy, a biopsy of one of the lesions was obtained.

**Figure 2:**
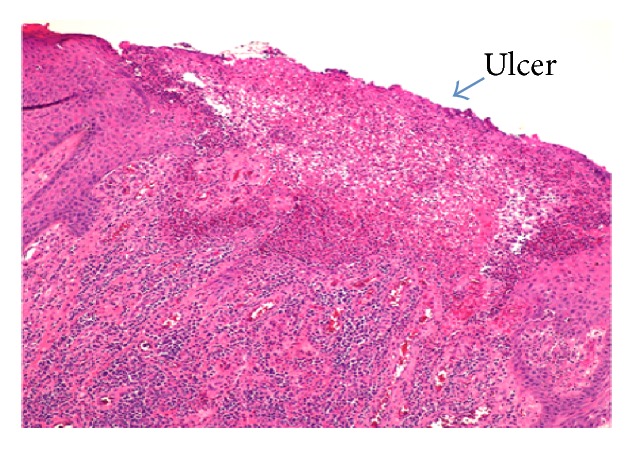
Histological sections of the lesion showing acute inflammatory and lymphoplasmacytic infiltrate in the dermis (hematoxylin-eosin (HE)), original magnification ×40).

**Figure 3:**
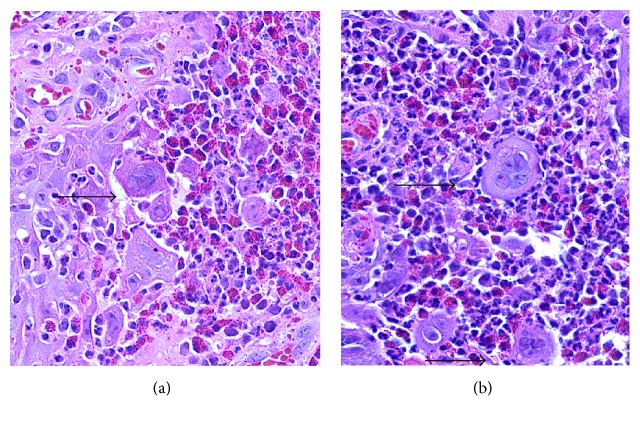
(a) Histopathologic appearance showing epithelial cell with viral inclusions. (b) Histopathologic appearance showing multinucleated cells (HE, original magnification ×400).

**Figure 4:**
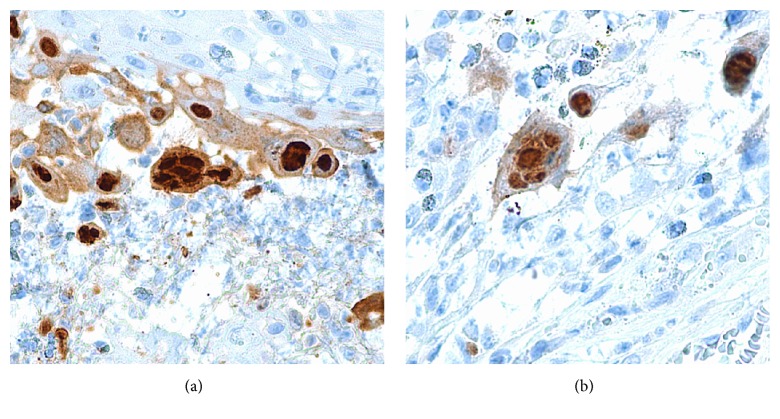
Cells from the biopsy specimen showing positive immunostaining for HSV-2 (a) and HSV-1 (b) (original magnification ×400).

**Figure 5:**
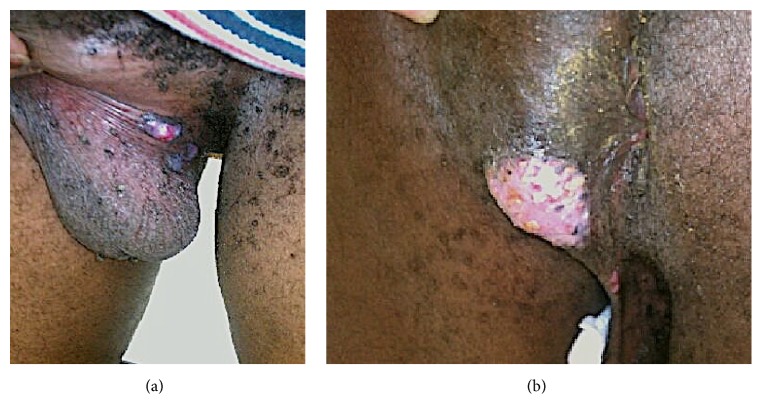
Appearance of pseudotumoral lesions on scrotum (a) and perineum (b) 11 days after starting leflunomide.

The biopsy specimen revealed an ulcerated epidermis with an associated acute inflammation and prominent lymphoplasmacytic infiltrate (Figure 2). Along the base of the ulceration were epidermal cells demonstrating viral cytopathic changes and multinucleated giant cells (Figures 3(a) and 3(b)). These cells stained positively with HSV-1 and HSV-2 immunostains (Figures 4(a) and 4(b)). Cytomegalovirus (CMV) immunostain, Epstein-Barr virus (EBER) in situ hybridization, and Gomori methenamine silver (GMS) and Fite stains were negative. Viral cultures of the lesions were negative. Two weeks into treatment with foscarnet there was no significant change in the size of the lesions. Based on the report of Henao-Martínez on the successful use of leflunomide in an HIV patient with HSV-2 proctitis [7], leflunomide 20 mg orally twice daily was then started; intravenous foscarnet and topical imiquimod were continued. Herpes virus was not cultured during this episode, so susceptibility testing could not be performed. The patient received 23 days of foscarnet, 14 days of topical imiquimod, and 11 days of leflunomide with approximately 80% reduction in the size of the perineal lesion compared to the size at the time of hospital admission (Figure 5). This initial response may have been due to the combination of foscarnet, imiquimod, and leflunomide. He was discharged on leflunomide 20 mg orally twice a day. The lesions continue to improve over time; after nine months of leflunomide, there was complete regression of the large perineal lesion and only two small ulcerations remained on the scrotum.

## 3. Discussion

The usual differential diagnosis for exophytic lesions in the anogenital area in the setting of HIV infection includes giant condyloma acuminatum, condyloma lata of secondary syphilis, mycobacterial lesions, squamous cell carcinoma, or lymphoma [6]. However, HSV infection can also present as hypertrophic or tumorous lesions [3, 5, 8–10]. The largest series has been published by Sbidian and coworkers, who described the clinical characteristics of ten HIV-infected patients with pseudotumors associated with HSV-2. In their series, at the time of pseudotumor diagnosis, the average CD4 count of the ten patients was 481 cells/*μ*L with a range of 165–632; all of the patients had undetectable HIV viral loads. For seven of the patients, there were multiple lesions. No coinfection with HSV-1 was observed in this series. Histopathologic exam of the lesions in each case showed a moderately dense dermal polytypic plasma cell infiltrate [3].

Sbidian et al. have proposed that HSV-associated pseudotumors arise from a dysregulated HSV antigen-driven immune reaction and further suggest that the low contribution of T cells to the lesional cellular infiltrate indicates a restricted functional defect of HSV-2-specific T cells. Thus, lesion hypertrophy and pseudotumor formation is a dysfunctional immune reconstitution event [3].

In six of eight cases in the Sbidian series in which susceptibility assays were performed, acyclovir resistance was observed. However, even in cases in which the viral isolate was susceptible, these pseudotumors persisted despite acyclovir treatment because drug penetration into these masses may be limited [3]. In their series, durable control was observed with HSV DNA polymerase inhibitors (acyclovir, cidofovir, and foscarnet) in only two of the ten cases, whereas the immunomodulators imiquimod and thalidomide afforded sustained response in five patients [3].

However, the use of thalidomide has several potential pitfalls. In addition to its well-known teratogenicity, thalidomide also carries a Food and Drug Administration Black Box warning for increased risk of thromboembolism. It may also cause peripheral neuropathy (which may be irreversible) in greater than 10% of patients. Due to its potential adverse effects, practitioners in the United States are required to undergo special training before prescribing thalidomide and adhere to a drug-specific Risk Evaluation and Mitigation Strategy [11]. Furthermore, failure with thalidomide in the treatment of hypertrophic HSV lesions has also been reported [5]. Topical imiquimod has also been used successfully in the treatment of hypertrophic and pseudotumoral genital herpes simplex infection refractory to acyclovir and foscarnet [6, 12]. However, the response of herpes pseudotumors to imiquimod may require several months of treatment [6].

Infections with herpes simplex virus-1 (most commonly orolabial) and herpes simplex virus-2 (usually genital) (HSV-1 and HSV-2) infections are typically responsive to acyclovir or its prodrugs. Herpes strains can become resistant to acyclovir, and HSV infection due to acyclovir-resistant virus is most commonly treated with foscarnet or cidofovir. Response of classic cutaneous herpes to foscarnet is relatively rapid (7–10 days) [13, 14]. However, foscarnet and cidofovir are potentially nephrotoxic and require intravenous administration. Hence for cases of herpes refractory to acyclovir, practical long-term treatment options are limited.

Leflunomide is an immunosuppressant used primarily in the treatment of rheumatoid and psoriatic arthritis. In 1999, Waldman and colleagues reported that leflunomide inhibited cytomegalovirus (CMV) by a mechanism distinct from other antivirals, by inhibiting viral capsid assembly [15]. Leflunomide was first used in the treatment of ganciclovir-resistant CMV infection in 2004 [16]. Morita et al. recently reviewed thirty cases of resistant CMV infection in transplant recipients treated with leflunomide; there were twenty successful treatments, five transient responses, and five failures [17]. Although the success rate was only 66.7%, ganciclovir-resistant CMV infection is a challenging problem in a highly immunocompromised population.

In 2001, it was demonstrated that leflunomide is also active in vitro against HSV-1 [18]. Clinical experience with leflunomide in the treatment of herpes virus infections is more limited than for CMV infection. Avery and coworkers reported that orolabial herpes improved in a transplant patient given leflunomide to treat CMV infection [16]. Henao-Martínez and colleagues reported that leflunomide successfully treated a case of acyclovir-resistant HSV-2 proctitis in an HIV patient [7]. In the case described herein, acyclovir resistance was not documented; however, the pseudotumoral lesions formed while on treatment doses of valacyclovir and were poorly responsive to foscarnet. Although resolution of the pseudotumors was slow, this case demonstrates a beneficial effect of leflunomide in a case of mixed HSV-1 and HSV-2 infection poorly responsive to conventional therapies. Furthermore, leflunomide is well tolerated over a long treatment duration [19]. This patient experienced no adverse effects to leflunomide and he continued to accrue CD4 cells during the treatment; after nine months of leflunomide treatment, his CD4 count increased from 399 cells/*μ*L to 418 cells/*μ*L.

In the treatment of HSV pseudotumors, leflunomide has dual mechanisms of action: the aforementioned antiviral activity and immunomodulation [20]. The proposed mechanism of leflunomide in the treatment of autoimmune disease is the inhibition of mitochondrial dihydroorotate dehydrogenase, which is involved in the synthesis of the RNA nucleotide uridine monophosphate. Thus, leflunomide interferes with cell cycle progression, thereby preventing the expansion of activated lymphocytes [21]. Pseudotumor formation in herpetic infections in HIV patients has been proposed to be an immune reconstitution phenomenon [3, 12]. This patient did have significant immune reconstitution over the two-year period prior to presentation; his CD4 count increased from 156 cells/*μ*L (9%) to 399 cells/*μ*L (22%) over that time.

Most of the cases of pseudotumor formation have been reported in association with HSV-2 infection [3]. This patient had a mixed HSV-1 and HSV-2 infection, which are relatively common. In a series of 183 genital samples from patients in Thailand, mixed infection was found in 18% and 14% of Thai and foreigner groups, respectively [22]. There are no obvious differences in the clinical course of mixed herpes infections compared to single HSV-1 or HSV-2 infections [23]. In one of the prior cases of HSV-associated pseudotumor, there was mixed HSV-1 and HSV-2 infection [10].

## 4. Conclusions

In conclusion, a patient with HIV infection presented with genital pseudotumor formation associated with HSV-1 and HSV-2 infection. The pseudotumors did not respond to treatment with acyclovir and foscarnet but showed near complete resolution with leflunomide, an agent with antiviral and immunosuppressant properties. Leflunomide deserves additional investigation for this application and should be compared to thalidomide in future studies.
